# Correspondence

**Published:** 1870-09

**Authors:** 


### Correspondence.

PONTIAC, MICH., September 27, 1870.

DR. J. N. HODGEN—*Dear Sir:* I have been disappointed in not seeing your article in the REGISTER, and consequently would like to ask a few questions:

*First.* How much Bismuth will be necessary in order to have plenty of die metal?

*Second.* Do you pour the metal into the impression, if so, do you use the common impression cup?

*Third.* What metal do you use for counter die? Pardon me for trespassing further upon your time, and believe me,

Yours, etc.,

J. A. HARRIS.

LEXINGTON, KY., September 29, 1870.

DR. J. A HARRIS—*Dear Sir:* I take pleasure in replying to your inquiries:

*First.* I give you my recipe for the improved die metal:

| | |
|---|---|
| R—Bismuth, | 8 lbs. |
| Lead, | 5 " |
| Tin, | 3 " |
| Antimony, | 2 " |

Melt together, stirring to mix thoroughly. You will discover that the antimony is the hardest to melt, and when you use it, do not heat sufficiently to melt it, but the fine crystals penetrating throughout the mass will hold the other metals in a plastic state for some time, and sufficiently so to allow you to dip your cast and take it out and redip and press it in further if necessary to improve your impression in the metal. This quantity will make about four dies, enough to have duplicates. I prefer to have a set to finish on—the first will be a little battered.

*Second.* You ask: "Do you pour the metal into the impression?" No, Fill your impression with plaster after using a paint of gum shellack, made with alcohol, and then oil it; trim so that it can be easily removed by slanting it. Have a ring of iron one-eighth inch thick will do by one inch deep, the size of a flask. Place this on paper, and pour in a little of your metal and wait until it stiffens so it will not run, then pour full, having it so little melted that it will hardly pour, dip in your cast and press it down sufficiently, then take it out before the steam injures the impression when it is damp. If the metal runs any, dip it in again, and press it as before, and so repeat it until the metal ceases to run. But if the cast recedes much in front, dry it to get a perfect fit. And when you dip it into the second ring, let it remain until cool enough to prevent breaking the metal, which is quite tender when hot. You should make a hole through your cast to let the air escape while pressing it down, and you can tack in a lead chamber piece before dipping if you wish—this will not melt. These I seldom make, as they do no good but for a few days, except to strengthen the plate, as I conceive.

Place on your dies another ring of same shape but smaller—this may be of hoop iron, with the ends in contact, but not fastened, and after you pencil the metal with whiting and water—have it thin, as soon as dry pour in the metal, so stiff that it will scarcely run, when cool separate them, swedge and strike up on the first, and finish on the second pair. I regard this as much easier than the zinc, and you will get as good fits, perhaps better.

To get up gold bands, take your impression after you have ground up your teeth, dip in the same way, you can make rings to suit the shape of cast.

P. S.—When your metal gets full of dirt, soot, etc., make it hot, and skim off the filth.

Yours respectfully,

J. N. Hodgen.

